# Band Structure Engineering in 2D Metal–Organic Frameworks

**DOI:** 10.1002/advs.202404667

**Published:** 2024-08-09

**Authors:** Simone Mearini, Daniel Baranowski, Dominik Brandstetter, Andreas Windischbacher, Iulia Cojocariu, Pierluigi Gargiani, Manuel Valvidares, Luca Schio, Luca Floreano, Peter Puschnig, Vitaliy Feyer, Claus Michael Schneider

**Affiliations:** ^1^ Peter Grünberg Institute (PGI‐6) Jülich Research Centre 52428 Jülich Germany; ^2^ Institute of Physics University of Graz Graz 8010 Austria; ^3^ Department of Physics University of Trieste Trieste 34127 Italy; ^4^ Elettra‐Sincrotrone Trieste S.C.p.A S.S. 14 km 163.5 Trieste 34149 Italy; ^5^ ALBA Synchrotron Light Source Barcelona 08290 Spain; ^6^ TASC Laboratory CNR–Istituto Officina dei Materiali (IOM) Trieste 34149 Italy; ^7^ Faculty of Physics and Center for Nanointegration Duisburg‐Essen (CENIDE) University of Duisburg‐Essen 47048 Duisburg Germany; ^8^ Department of Physics and Astronomy UC Davis Davis CA 95616 USA

**Keywords:** angle‐resolved photoelectron spectroscopy, band structure engineering, density functional theory, molecular ligand, single‐layer metal–organic framework, transition metal, 2D materials

## Abstract

The design of 2D metal–organic frameworks (2D MOFs) takes advantage of the combination of the diverse electronic properties of simple organic ligands with different transition metal (TM) centers. The strong directional nature of the coordinative bonds is the basis for the structural stability and the periodic arrangement of the TM cores in these architectures. Here, direct and clear evidence that 2D MOFs exhibit intriguing energy‐dispersive electronic bands with a hybrid character and distinct magnetic properties in the metal cores, resulting from the interactions between the TM electronic levels and the organic ligand π‐molecular orbitals, is reported. Importantly, a method to effectively tune both the electronic structure of 2D MOFs and the magnetic properties of the metal cores by exploiting the electronic structure of distinct TMs is presented. Consequently, the ionization potential characteristic of selected TMs, particularly the relative energy position and symmetry of the 3d states, can be used to strategically engineer bands within specific metal–organic frameworks. These findings not only provide a rationale for band structure engineering in 2D MOFs but also offer promising opportunities for advanced material design.

## Graphical abstract

The study presents a promising approach to manipulate the band structure of 2D MOFs, which exhibit intriguing energy‐dispersive hybrid electronic bands resulting from the interaction between TM electronic levels and organic ligands π molecular orbitals. The control over energy level position and band dispersion of hybrid states is achieved by selecting the metal while utilizing the same molecular ligand.

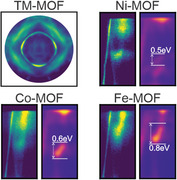

## 1 Introduction

Gaining precise control over matter, down to the atomic level, is an essential requirement in the attempt to miniaturize devices for modern applications. Consequently, one focus of current research is directed toward materials, typically inorganic, constructed through the layer‐by‐layer stacking of individual atomic layers. Such layered materials, commonly referred to as 2D materials, have strong in‐plane bonding between atoms, while the individual layers are held together by weak van‐der‐Waals (vdW) interactions.^[^ ^1^, ^2^, ^3^ ^]^

A possible alternative to these inorganic 2D materials relies on molecular‐based frameworks supported on bulk substrates.^[^ ^4^, ^5^, ^6^, ^7^, ^8^, ^9^ ^]^ A wide range of periodic and robust low‐dimensional molecular architectures, also known as 2D MOFs, have already been fabricated.^[^ ^5^, ^7^, ^10^, ^11^ ^]^

Their electronic properties are governed by the interaction between metal ions coordinated by organic ligands. Careful considerations allow the realization of exciting functional properties like magnetic ordering,^[^ ^9^, ^12^ ^]^ spin selective tunneling,^[^ ^10^ ^]^ and high electric conductivity.^[^ ^11^, ^13^ ^]^ or even superconductivity.^[^ ^14^ ^]^ For example, clear evidence of long‐range ferromagnetic order in a 2D MOF has been reported.^[^ ^15^ ^]^

Besides their magnetic activity, MOFs may also exhibit energy‐dispersive electronic features.^[^ ^16^, ^17^ ^]^

In molecular‐based devices, research efforts in band structure engineering have primarily been focused on disordered and weakly crystalline molecular films constructed from various molecular precursors.^[^ ^18^, ^19^ ^]^ In such systems, the behavior of the charge carriers can effectively be described by the localized polaron concept.^[^ ^18^ ^]^ In current electrical and optical technologies, there is a growing demand not only for tuning the energy structure in organic‐based materials but also for the miniaturization of corresponding devices to nearly atomically thin dimensions. This directs the attention to single‐layer MOFs. However, band structure engineering in 2D MOFs has yet to be explored in depth.

Recently, we have fabricated metal–organic structures on noble metal surfaces using 1,2,4,5‐tetracyanobenzene (TCNB) and co‐deposited Ni atoms. Based on scanning tunneling microscopy (STM) investigations, we have observed that, on Au(111), two different Ni‐(TCNB)_x_ (x = 2, 4) phases form depending on the amount of Ni, while, on Ag(100), the pristine TCNB layer is immediately converted into an extended Ni‐(TCNB)_2_ phase.^[^ ^20^ ^]^ Interestingly, energy‐dispersive hybrid states emerged from the interaction between Ni 3d states and the π‐symmetric TCNB frontier molecular orbitals on both substrates, as confirmed by valence band spectroscopy and density functional theory (DFT) calculations. These findings encourage further exploration of such phenomena for potential band structure engineering in 2D MOFs, as required by modern electronics and photonics applications.

In the present work, we elucidate the electronic and magnetic properties of 2D MOFs stabilized on an Ag(100) substrate. We focus specifically on affecting the energy levels and band dispersion by the substitution of different central metal cores, namely Fe, Co, and Ni while maintaining the same TCNB precursor. A state‐of‐the‐art photoemission momentum microscope is used to probe the photoelectron momentum distribution of the metal‐ligand states. We observe that the selection of the metal atom is crucial in the tuning of the electronic levels of the hybrid states since different TMs possess a distinct electronic structure and 3d level alignment. These results are further supported by theoretical investigations using DFT calculations. The combined effort of these methodologies allows the rationalization of the engineering of electronic bands in 2D MOFs, caused by the differences in the electronic structure, a phenomenon not observed up to now.

## 2 Results

**Figure 1:**
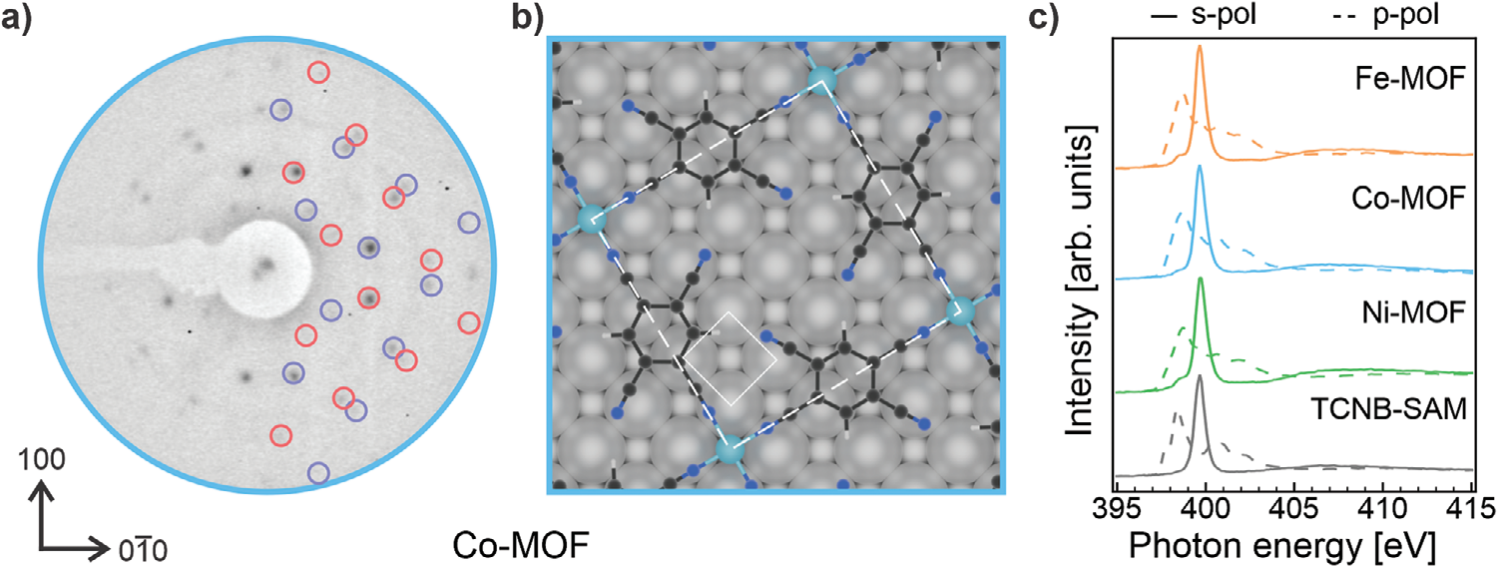
The structural rearrangement of the TCNB layer on Ag(100) upon TM intrusion leads to a TM‐MOF architecture, as confirmed by the experimental LEED patterns and the linear dichroism in the NEXAFS spectra measured across the N K‐edge. a) Experimental LEED pattern acquired with incident electron beam energy of 20 eV for the Co‐MOF; the red and blue circles, corresponding to two rotational domains, indicate the simulated LEED patterns. b) Real‐space structure representation of the relaxed structure for the Co‐MOF on Ag(100). c) NEXAFS spectra acquired across the N K‐edge with s‐ (solid line) and p‐polarized light (dashed line) for all the TM‐MOFs and the pristine TCNB‐SAM interface.

We synthesize the TM‐(TCNB)_2_ MOFs by depositing TCNB molecules onto an Ag(100) substrate until a saturated molecular array is reached, as confirmed by low‐energy electron diffraction (LEED) measurements (see **Figure** **1**a; Figure S1, Supporting Information). In the initial step, the interaction between the molecules and the substrate governs the geometrical arrangement of the molecular species, forming a TCNB‐based self‐assembled monolayer (TCNB‐SAM). Once the coverage reaches saturation, the LEED pattern progressively sharpens and remains unchanged with the addition of more molecules, indicating the absence of a second‐layer formation. Additionally, the relative intensity of spots associated with both the substrate and molecular superstructure remains constant after saturation is reached. Subsequently, TM atoms (Fe, Co, or Ni) are co‐adsorbed onto this interface while maintaining the sample at room temperature. Notably, the long‐range order of the molecules is maintained after the intrusion of the TMs into the molecular array. The LEED measurements reveal a consistent pattern across all studied MOFs, which we show for the Co‐(TCNB)_2_ in Figure 1a and extensively in Figure S1 (Supporting Information). The structure is characterized by an epitaxial matrix (4, 1; −1, 4), consistent with the previous STM measurements reported for the Ni‐(TCNB)_2_.^[^ ^20^ ^]^ Starting from this matrix, we confirm the structural analogy of the three MOFs by simulating the LEED patterns. The resulting patterns, reported in Figure 1a and Figure S1 (Supporting Information) as blue and red circles, corresponding to two rotational domains, are superimposed on the right half of the experimental LEED figures. The perfect match between the simulated and experimental data for all three cases confirms the structural analogy. For illustrative purposes, the structural model of the Co‐(TCNB)_2_ is reported in Figure 1b, with the TMs of the interconnected Co‐(TCNB)_2_ units coordinated in a square planar environment in between the TCNB linkers. Analogous models for the Ni‐(TCNB)_2_ and Fe‐(TCNB)_2_ are shown in Figure S2 (Supporting Information). From now on, these structures will simply be referred to as Fe‐MOF, Co‐MOF, and Ni‐MOF.

Further insight into the geometrical properties of these networks is gained from near‐edge x‐ray absorption fine structure (NEXAFS) experiments. As reported in Figure 1c, we measured NEXAFS across the N K‐edge with the impinging x‐ray electric fields of the radiation oriented parallel (s‐polarization, solid line) and closely normal (p‐polarization, dashed line) to the surface, thereby probing the σ*‐ and π*‐ absorption resonances of our systems, respectively.^[^ ^21^, ^22^ ^]^ As evident from Figure 1c, all three interfaces, i.e., Fe‐, Co‐, and Ni‐MOF, show very similar spectra. More specifically, the π*‐symmetry resonance at a photon energy of ≈399 eV, associated with the CN groups of TCNB, is only observed with p‐polarized light, while a sharp σ* resonance at ≈400 eV is manifested with s‐polarized light.^[^ ^23^, ^24^ ^]^ The linear dichroism strongly suggests a flat on‐surface orientation of the CN groups and, thus, a flat adsorption structure of the whole TCNB molecule in MOFs. Note that we have already detected the same linear dichroism in the N K‐edge NEXAFS spectra of the pristine TCNB layer on Ag(100) (see Figure 1c), suggesting that, upon the TM coordination, the initially flat geometry of the organic ligands is preserved. The dichroic behavior observed in the π*‐symmetry resonances of the C K‐edge spectra of MOFs measured with p‐ and s‐polarized light, attributed to the benzene ring of TCNB,^[^ ^21^, ^22^, ^23^ ^]^ further confirms the flat geometrical orientation (see Figure S3, Supporting Information). Besides providing geometrical insight, NEXAFS analysis is also sensitive to the bonding environment of the absorbing atoms.^[^ ^22^ ^]^ A similar fine structure in the N and C K‐edge spectra obtained from the three different MOFs further confirms the presence of similar coordination environments for the cyano groups of the ligands in the studied systems.

From our LEED and NEXAFS experiments, we therefore confirm that the geometrical structure of our MOFs is not TM‐dependent. However, we expect the magnetic properties on the TM centers and the electronic structures of the MOFs to be significantly different between the different MOFs due to the initially diverse electronic configurations of the metal´s d‐states: Fe (d^8^), Co (d^9^), and Ni (d^10^).^[^ ^25^, ^26^, ^27^, ^28^, ^29^ ^]^

**Figure 2:**
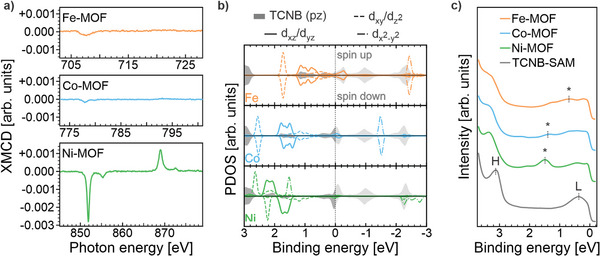
Magnetic and electronic structure of the TM‐MOFs determined from experiment and theory. a) XMCD spectra obtained at normal incidence geometry (T≈2 K, B = 6 T). b) Simulated DOS projected onto Ni 3d and TCNB 2p_z_ states. c) Valence band spectra obtained with an excitation photon energy of 30 eV and p‐polarized light; “L‐”, “H‐” and “*‐” refers to the filled LUMO, HOMO, and hybrid states, respectively. The following color code has been chosen: green, cyan, and orange for the Ni‐, Co‐, and Fe‐MOFs and gray for the TCNB‐SAM, respectively.

To further explore this hypothesis, the magnetic properties of the metal centers are probed via L_3,2_‐edge x‐ray magnetic circular dichroism (XMCD) experiments. The corresponding dichroic signals, i.e., XMCD data, for the three systems, are shown in **Figure** **2**a. It is defined by the difference of the two x‐ray absorption spectra (XAS) acquired with left (C^−^) and right circularly (C^+^) polarized light across the L_3_‐ and L_2_‐edges of the TMs. The raw XAS data measured at a magnetic field B = 6 T along the wave vector of the incident photon are depicted in Figure S4 (Supporting Information). The XMCD spectrum of Ni‐MOF, in particular the resonance at lower photon energy, shows a strong dichroic signal at the L_3,2_‐edge. This observation points toward a configuration with unpaired electrons at the Ni center and supports the stabilization of a Ni(I) (d^9^) ion within the Ni‐MOF as proposed previously.^[^ ^20^ ^]^ Contrary to the Ni‐MOF, the XMCD data collected for the Co‐ and the Fe‐MOFs show very low dichroic signals, suggesting the stabilization of both the Co and Fe centers in low‐spin electronic configurations, with the electrons paired in the occupied d‐shells. Therefore, we expect that the MOFs manifest as Co(I) (d^8^) and Fe(II) (d^6^) ions.

To elucidate this point, we carried out DFT calculations using the relaxed structure model reported in Figure 1b and Figure S2 (Supporting Information). It is worth noting that, by exploiting the commensurability of the MOFs with the Ag(100) unit cell, we can also include the substrate in our repeated slab model of the interface. We optimize the starting geometry employing the PBE‐GGA exchange‐correlation functional with a DFT‐D3 vdW‐correction as well as a DFT+U self‐interaction error correction for the strongly correlated d‐orbitals in the transition metal (see “Experimental section” for more details).

The magnetic configuration can be inferred from Figure 2b which shows the spin‐resolved density of states (pDOS) projected onto the d‐states of the TM center (colored lines) and the p_z_ states of TCNB (shaded gray area) for all three MOFs under consideration. Note that we use a positive sign convention for the binding energy (BE) of the occupied states. From the spin‐splitting in the d‐orbitals of the transition metals, we can confirm a spin‐polarized state for Ni and a spin‐unpolarized state for Co, in full agreement with the experimental XMCD data discussed above. The situation for the Fe is more involved. Here, we find two stable configurations, where the spin‐polarized one is lower in total energy by −0.48 eV, but the experimental XMCD data indicate the spin‐unpolarized configuration to be favored. Therefore, we will focus the following discussion solely on the spin‐unpolarized solution.

From a projection onto molecular orbitals (see Figure S5, Supporting Information), we attribute the TCNB contribution around the Fermi edge to the former lowest unoccupied molecular orbital (LUMO) of TCNB, while the peak ≈+3 eV of BE consists of the highest occupied molecular orbital (HOMO) and contributions from the lower‐lying molecular orbitals. The energy level alignment suggests a charge transfer into the TCNB LUMO at the interface. On the one hand, as we will discuss below, this is partly due to the interaction between the TM central atom and the TCNB linkers. On the other hand, a partial occupation of the TCNB LUMO is already predicted for the pristine TCNB‐SAM interface suggesting also an additional charge transfer from the Ag substrate to the LUMO of TCNB (see Figure S6, Supporting Information). Most notably, however, our investigation reveals a newly arising state in the energy range of 0.6–1.1 eV (Fe), 1.4–1.8 eV (Co), and 1.6–2.4 eV (Ni), respectively (see Figure 2b). As can be seen in the projected DOS, it shares contributions from states of both the frontier orbitals of the TCNB molecules and the central transition metal atoms. We, thus, label them as hybrid states.

To understand the nature of these hybrid states, we may briefly reflect on the local symmetry of our metal–organic systems. Neglecting the influence of the substrate, we can regard the TCNB molecules as a square planar coordination environment around the TM central atoms exhibiting a D_4h_ point group. From symmetry considerations, we then expect a splitting of the five‐fold degenerate d‐orbitals into a set of three distinct energy levels ($d_{xy}$, $d_{z^{2}}$, $d_{x^{2}-y^{2}}$) and one two‐fold degenerate level ($d_{xz}$, $d_{yz}$). Among these, only $d_{xz}$ and $d_{yz}$ share the π‐symmetry of the TCNB HOMO and LUMO and, consequently, can hybridize with the frontier molecular orbitals of TCNB. Moreover, the specific energy ordering of the d‐states can be rationalized from crystal field theory, which considers the electrostatic repulsion between electrons in the metal with the coordination environment. Simply assuming point charges on the TCNB molecules already suffices to correctly predict the ordering for the unhybridized d‐states of $d_{xy}$, $d_{z^{2}}$, $d_{x^{2}-y^{2}}$ from lowest to highest in energy. The $d_{x^{2}-y^{2}}$ orbital is the highest in energy because it lies in the plane of the metal ion and aligns along the x and y axes. Consequently, its lobes point directly toward the ligands in a square planar arrangement, leading to maximum electron‐electron repulsion. This interaction significantly raises the energy of the $d_{x^{2}-y^{2}}$ orbital compared to the other d‐orbitals, positioning it even higher in energy than the LUMO, whose energy is low due to the high electron affinity of TCNB.^[^ ^30^, ^31^ ^]^

The calculated spin configuration of the TM atom also reveals how much charge is transferred from the central metal ion to the TCNB molecules upon coordination of the TM. It is worth noting that this charge transfer already exists in calculations for the freestanding MOF layer without substrate and, thus, can be considered independent from the previously mentioned charge transfer from Ag to TCNB (see Figure S6, Supporting Information). In the case of Ni and Co, the charge rearrangement generated by the square planar crystal field results in the $d_{x^{2}-y^{2}}$ having the highest energy among all the d orbitals, as mentioned above, and being positioned above the LUMO of TCNB. This induces a (I) oxidation state for both the Ni and Co ions and the presence of one and zero electrons in the $d_{x^{2}-y^{2}}$ orbital of the two ions, respectively. Formally, the TM donates a charge, thereby pushing the former LUMO below the Fermi edge and leaving the metal in an (I)‐oxidation state. As for Fe, the $d_{x^{2}-y^{2}}$ orbital holds no electrons, meaning that any charge transfer has to involve the next‐in‐line $d_{xz/yz}$ states. However, simultaneously, we have found these states to hybridize with the frontier orbitals of TCNB, changing the simple picture of integer charge transfer from the d‐states to a more covalent nature. Evidence for this can be seen in the increased contribution of the $d_{xz/yz}$ at the position of the LUMO right above the Fermi edge and the, therefore, missing charge in the $d_{xz/yz}$ orbitals. The degeneracy of the $d_{xz/yz}$ orbitals introduces complexity, allowing for the observation of a spin‐unpolarized solution experimentally, despite the calculation indicating that the spin‐polarized configuration has a lower total energy. Thus, based on our analysis, we attribute the following formal electronic configurations to the TMs in our MOF systems: ($d_{xy}$)^2^ ($d_{xz/yz}$)^4^ ($d_{z^{2}}$)^2^ ($d_{x^{2}-y^{2}}$)^1^ for the Ni‐MOF, ($d_{xy}$)^2^ ($d_{xz/yz}$)^4^ ($d_{z^{2}}$)^2^ ($d_{x^{2}-y^{2}}$)^0^ for the Co‐MOF and ($d_{xy}$)^2^ ($d_{xz/yz}$)^4^ ($d_{z^{2}}$)^0^ ($d_{x^{2}-y^{2}}$)^0^ for the Fe‐MOF. These electronic arrangements in the 3d orbitals are in accordance with theoretical investigations and XMCD findings.

The proposed configurations of the TM within the MOFs are further confirmed by the linear dichroism behavior observed in the NEXAFS spectra measured across the L_3_‐edge in s‐ and p‐polarization (see Figure S7, Supporting Information). The reported spectra show, in fact, similar features to the one reported previously for metal–organic systems,^[^ ^32^, ^33^, ^34^, ^35^ ^]^ confirming the square planar coordination environment and the observed TMs electronic configurations.

We test the theoretically predicted electronic structure by performing valence band (VB) photoemission spectroscopy. We first focus on the momentum‐integrated VB spectra reported in Figure 2c for the TCNB‐SAM system and the three MOF interfaces. In this case, the photoemission intensity is integrated over a 2D momentum map (k_||,x_, k_||,y_: ≈±2 Å^−1^) while scanning the BE. The VB spectrum of the pristine TCNB layer on Ag(100) manifests two prominent peaks at BE values of 0.35 and 3.10 eV. The comparison with our calculations suggests that the spectral feature centered at BE 3.10 eV is associated with the HOMO of the TCNB, while the feature closer to the Fermi level, at BE 0.35 eV, can be attributed to the LUMO. In agreement with the theory, the occupation of the former LUMO is already observed experimentally in the pristine TCNB layer, and, thus, confirms a charge donation from the substrate to the adsorbed molecules. Similar behavior has also been observed for tetracyanoquinodimethane (TCNQ) when deposited on the same substrate.^[^ ^36^ ^]^

When considering the TM‐MOFs, the main difference induced by the addition of the TM is the appearance of new features in the region between the spectroscopic HOMO and LUMO fingerprints. Although the HOMO peak undergoes small BE shifts in the presence of the TMs, the new features depend significantly on the specific TM center. In fact, comparing the three spectra, we find the peak maxima of the new states (*) shifting from 1.5 eV (Ni) to 1.3 eV (Co) and 0.7 eV (Fe). Hence, our data strongly suggest that the metal–organic coordination is responsible for the emergence and the energy position of these new states. This finding is consistent with the calculated pDOS discussed in Figure 2b leading us to attribute the experimental features to the hybrid states formed between the TM 3d states and the ligand molecular orbitals. Finally, we observe that the VB spectra acquired with p‐polarized light show a higher intensity for the hybrid state features compared to the s‐polarization case (see Figure S8, Supporting Information). This difference is indicative of the predominantly π‐symmetry character of these new hybrid states.

**Figure 3:**
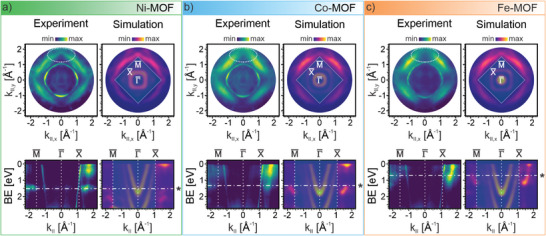
ARPES measurements and simulations for the 2D MOFs on Ag(100) substrate. *Top*: experimental (left) and simulated (right) constant energy 2D momentum maps (k_||,x_, k_||,y_) of the newly formed hybrid states at BE of a) 1.50 eV b) 1.30 eV and c) 0.70 eV. *Bottom*: experimental (left) and simulated (right) valence band maps represented as BE versus momentum cuts along the $\bar{M}-\bar{\Gamma }-\bar{X}$ direction of the supporting surface. The experimental data are obtained with excitation photon energy of 30 eV and p‐polarized light. The Ag(100) surface Brillouin zone is shown on the simulated maps. The cuts and maps refer to the three studied systems: a) Ni‐MOF, b) Co‐MOF, c) Fe‐MOF. The stars (*) indicate the hybrid states. The shaded boxes in the simulated cuts and maps refer to the areas where the photoemission intensity from the Ag(100) cannot be simulated correctly with our five‐layer slab calculations.

For a more in‐depth understanding of the electronic structures of the MOFs, we will continue by analyzing our photoemission data of the VB spectra in more detail. Our photoemission electron microscope allows us to efficiently measure the photoemission intensity over a broad range of emission angles. Angle‐resolved photoemission spectroscopy (ARPES) is commonly employed to elucidate the energy level alignment of self‐assembled molecular networks.^[^ ^16^, ^17^, ^20^, ^36^ ^]^ It is also worth noting that our specific setup minimizes the radiation damage often observed and critical for organic‐based systems,^[^ ^37^, ^38^ ^]^ making it ideally suited for investigating MOFs (see “Experimental section” for details). By collecting the intensity over an energy range down to the photoemission onset of the Ag d‐bands (at ≈3.5 eV), we obtain a 3D data cube of photoemission intensities as a function of k_||,x_, k_||,y_, and photoelectron BE, for each of the interfaces. From the experimental data, we extract the angle‐resolved intensity at constant BE to yield the so‐called momentum maps (k_||,x_, k_||,y_, BE = constant) shown at the top left of the three panels in **Figure** **3**a–c. For each interface, we display the momentum maps at the energy corresponding to the maximum intensity of the presumed hybrid state in the VB spectra, namely at 1.5 eV (Ni‐MOF), 1.3 eV (Co‐MOF), and 0.7 eV (Fe‐MOF). The three interfaces manifest momentum maps with similar features. Indeed, irrespective of the interface, the maps are characterized by four lobes centered at a k_||_‐radius of ≈1.5 Å^−1^. The similar emission patterns again hint at the similar nature of the states for all three TM‐MOFs.

Within the framework of photoemission orbital tomography,^[^ ^39^, ^40^, ^41^ ^]^ the comparison of measured k‐maps with simulated ones allows us to interpret the photoemission features in terms of specific molecular states. To this end, to the right of each experimental map in Figure 3a–c, we show the simulated momentum maps corresponding to the energy region of the metal‐ligand hybrid state from our calculated pDOS (see Figure 2b). The excellent agreement between the experimental maps and their theoretical counterparts unambiguously links them to emissions from the arising hybrid state. Interestingly, a fine structure in the experimental patterns such as the sharp low‐intensity lines around k_||_ ≈1.9 Å^−1^ as well as the missing intensity in the middle of the lobes at ≈1.5 Å^−1^, highlighted in the experimental maps by the dashed oval lines in Figure 3, is most pronounced for the Ni‐MOF. This observation could be partly attributed to the distinct energy separation of the hybrid state at this interface from any overlapping TCNB features (see Figure 2c), which would smear out the experimental maps.

The surface Brillouin zone of the substrate is indicated in the simulated maps. As can be seen from the comparison to the reference image of the pure Ag(100) surface momentum map (see Figure S9, Supporting Information), the emissions within the Ag(100) Brillouin zone are solely due to the substrate. Note that with our five‐layer slab calculations, we will not be able to quantitatively account for these intensities.^[^ ^42^ ^]^

The variation of the hybrid states in k‐space versus BE can equally be appreciated by cutting our 3D photoemission data stacks along the high‐symmetry direction $\bar{M}-\bar{\Gamma }-\bar{X}$ of the surface Brillouin zone. The resulting experimental band maps are depicted for the three TM‐MOFs in the bottom row of Figure 3a–c. The bands of the hybrid states exhibit clear dispersion in energy, providing further evidence that our metal–organic frameworks have indeed formed extended structures. To compare the energy dispersion of the hybrid states, we integrate the photoemission intensity of the k‐maps within a momentum space ring with k_||_ radius spanning between 1.4 and 1.6 Å^−1^, and we plot it as a function of the BE (for details on our analysis, see Figure S10, Supporting Information). Analyzing such obtained intensity curves, we estimate an increase of the band dispersion in energies from ≈0.5 eV (Ni), to ≈0.6 eV (Co) and ∼0.8 eV (Fe) for the three TM‐MOFs. This observation could be attributed to the specificities of hybridization in the TM‐MOFs. As discussed above, Ni and Co donate integer charges from $d_{x^{2}-y^{2}}$, shifting the LUMO below the Fermi edge, leaving the metal in an (I)‐oxidation state. In contrast, Fe has no electrons in $d_{x^{2}-y^{2}}$. Consequently, in Fe‐MOF, $d_{xz/yz}$ is involved in the charge transfer, hybridizing with the frontier orbitals of TCNB, thereby changing the simple picture of integer charge transfer from the d‐states to a more covalent nature.

For comparison, Figure 3a–c (bottom row) display also the simulated ARPES band maps next to the experimental data. The trend of the increasing energy dispersion, its k‐dependence as well as its energy position is fully reproduced in the theoretical data sets. This leads us to the conclusion that both the band dispersion and energy position, of a 2D MOF hybrid state, can be selectively controlled by the electronic configuration of the transition metal and its metal–organic interaction with the molecular ligand.

## 3 Conclusion

In summary, our study presents a promising pathway for manipulating the band structure of 2D MOFs. Through systematic exploration of the geometric and electronic properties of these materials, we have demonstrated how the relative energy positions of TM 3d states influence their hybridization with organic linker orbitals. The energy level position of the hybrid states and, most importantly, the band dispersion can be precisely controlled through the choice of the metal core while using the same molecular ligand. This suggests that 2D MOFs inherently possess multifunctional electronic and magnetic properties.

Indeed, the emergence of additional electronic hybrid states, which contain contributions from TMs and molecular ligands, in the electronic structure of the MOFs provides more pathways for optical transitions to states above the Fermi level.^[^ ^43^ ^]^ These aspects, together with the wider bandwidths, become important for applications involving light absorption and emission,^[^ ^44^ ^]^ such as in photovoltaics.^[^ ^45^ ^]^ or luminescent materials.^[^ ^46^, ^47^ ^]^ Furthermore, the ability to finely tailor the electronic structure in TM‐MOFs and harness redox‐active TM centers makes these systems a promising new class of photocatalysts,^[^ ^48^ ^]^ offering distinct advantages over the conventional metal oxide ones,^[^ ^49^ ^]^ and electrocatalysts.^[^ ^50^, ^51^ ^]^ Finally, the TM cores, possessing specific spin characteristics, determine the MOF magnetic properties as a whole, due to superexchange‐mediated interactions that can take place between TM ions through organic linkers.^[^ ^52^, ^53^ ^]^ This is particularly suitable for further exploring molecular spintronics applications.^[^ ^54^ ^]^

These insights pave the way for integrating such phenomena into future electronic and photonic devices utilizing 2D MOFs, offering exciting prospects for advanced material design pathways.

## 4 Experimental Section

### Sample Preparation

The Ag(100) single crystal was cleaned by repeated cycles of Ar^+^ sputtering followed by subsequent annealing to 800 K. TCNB was evaporated from a Knudsen‐type evaporator at 373 K onto the substrate kept at 300 K. We observed that TCNB does not form a second layer on Ag(100). Transition metals were evaporated from the e‐beam evaporator (FOCUS) operated at ion fluxes ranging from 5 to 10 nA (rate <0.1 Å min^−1^ on the sample). The structural rearrangement of TCNB was influenced by the amount of metal, providing control over the formation of the metal–organic phase using the LEED method. The LEED method was utilized to tune the deposition times for TMs within the multi‐technique approach presented in this study.

### ARPES Measurements

The ARPES experiments were performed at the NanoESCA beamline of the synchrotron light source Elettra in Trieste, Italy using an energy‐filtered photoelectron emission microscope (PEEM).^[^ ^55^ ^]^ By varying the kinetic energy of the photoelectrons using the PEEM, a 3D (k_||,x_, k_||,y_ vs BE) data stack consisting of 2D (k_||,x_ vs k_||,y_) constant BE momentum maps can be collected. A photon energy of 30 eV (p‐polarization) was used for the characterization of the 2D MOFs. The photon beam was incident at an angle of 65° relative to the surface normal. All the measurements were conducted at a pressure below 1 × 10^−10^ mbar keeping the sample at 90 K. The sample cooling was achieved by an open‐cycle cryostat (Janis ST‐400). The temperature is measured by a silicon diode (Lake Shore DT‐670E‐BR) at the sample holder. The total energy resolution (including both analyzer and beamline contributions) was 100 meV, while the momentum resolution of the PEEM was ± 0.05 Å^−1^. During the measurements, the sample was rastered to prevent damage induced by the photon beam and ensure the acquisition of high‐quality data. The sample manipulator has six degrees of freedom built on a SMARPOD motion system (SmarAct GmbH).

### NEXAFS Measurements

NEXAFS measurements were performed at the ALOISA beamline of the synchrotron light source Elettra in Trieste, Italy.^[^ ^56^ ^]^ NEXAFS spectra were collected by partial electron yield mode with a channeltron, equipped with a repelling grid polarized at a negative bias. The polarization was changed from Transverse Electric (s‐polarization) to Transverse Magnetic (almost p‐polarization) by rotating the sample around the photon beam axis at a constant grazing angle of 6° (i.e., without variation of the photon beam footprint on the sample). The normalization and energy calibration protocol for the absorption spectra collected is described in ref. [57] The NEXAFS experiment was performed at a pressure below 1 × 10^−10^ mbar while keeping the sample at 300 K.

### XMCD Measurements

XMCD experiments were conducted at the BOREAS beamline at the ALBA synchrotron.^[^ ^58^ ^]^ The magnetic field and temperature used for XMCD data were B = 6 T and T = 2 K. Measurements were taken at the Ni, Co, and Fe L_2,3_ edge, with the magnetic field fixed in the direction of the incident light. The spectra were collected by the total electron yield mode. Angle‐dependent measurements were carried out by rotating the sample about a vertical axis perpendicular to the synchrotron orbital plane, thereby varying the incidence angle between the x‐ray beam (and therefore the magnetic field) and the substrate normal. To avoid beam damage, the sample was continuously moving to access nonilluminated fresh sample spots.

### Theoretical Methods

We performed ground state density functional theory (DFT) calculations using the Vienna Ab initio Simulation Package (VASP), version 6.4.1 on the Vienna Scientific Cluster 5 (VSC‐5) and 5.4.4 on VSC‐4.^[^ ^59^, ^60^ ^]^ The exchange‐correlation effects were treated using the Perdew‐Burke‐Enzerdorf generalized gradient approximation (PBE‐GGA),^[^ ^61^ ^]^ along with a Grimme D3 vdW‐correction with Becke–Johnson damping.^[^ ^62^ ^]^ Within this GGA‐type exchange‐correlation functional, the inclusion of a self‐interaction error correction for the strongly localized d‐orbitals in the transition metals is crucial for an accurate representation of the hybridization observed in the experiments. Therefore, we introduced an effective Hubbard‐U parameter of 3 eV using the Dudarev ansatz.^[^ ^63^ ^]^ All calculations were performed in a spin‐unrestricted formalism to allow the system to relax into the energetically most favorable spin configuration. In the case of Co‐MOF, a spin‐restricted calculation, not shown here as well, showed a difference in total energy of 0.5 meV.

Starting with the experimentally determined structures, we fully relaxed all systems until all atomic forces were below 0.01 eV Å^−1^. We modeled the interface in the repeated slab approach with a 15 Å vacuum layer, adding a dipole layer within the vacuum region to address the electric field discrepancy between either side of the slab.^[^ ^42^ ^]^ The bulk of the silver substrate was modeled with a total of five layers, with relaxation permitted only in the top two layers during geometry optimization. The first Brillouin zone was sampled using a Γ‐centered 8 × 8 × 1 grid. For simulating the photoelectron distribution, the photoemission process was approximated as a one‐step process, where the final state was treated as a plane wave. Additionally, we included a damping of the substrate emissions according to ref. [64] of γ =  0.5 Å^−1^.

## Conflict of Interest

The authors declare no conflict of interest.

## Supporting information

Supporting Information

## Data Availability

The data that support the findings of this study are available on request from the corresponding author. The data are not publicly available due to privacy or ethical restrictions.
