# Supplementary material for: Band Structure Engineering in 2D Metal–Organic Frameworks
Source: Adv Sci (Weinh). 2024 Aug 9;11(38):2404667. doi: 10.1002/advs.202404667 (PMC11481395; doi:10.1002/advs.202404667)
Supplement: Supplementary file 1 — Supporting Information [file ADVS-11-2404667-s001.docx]

Supporting Information

Band Structure Engineering in 2D Metal-Organic Frameworks

Simone Mearini*, Daniel Baranowski, Dominik Brandstetter, Andreas Windischbacher, Iulia Cojocariu, Pierluigi Gargiani, Manuel Valvidares, Luca Schio, Luca Floreano, Peter Puschnig*, Vitaliy Feyer* and Claus Michael Schneider

S. Mearini, D. Baranowski, V. Feyer and C. M. Schneider

Peter Grünberg Institute (PGI-6), Jülich Research Centre, 52428 Jülich, Germany

* E mail: s.mearini@fz-juelich.de, v.feyer@fz-juelich.de

D. Brandstetter, A. Windischbacher, P. Puschnig

Institute of Physics, University of Graz, 8010 Graz, Austria

* E mail: peter.puschnig@uni-graz.at

I. Cojocariu

Physics Department, University of Trieste, 34127 Trieste, Italy

Elettra – Sincrotrone Trieste S.C.p.A, S.S. 14 km 163.5, 34149 Trieste, Italy

P. Gargiani, M. Valvidares

ALBA Synchrotron Light Source, 08290 Barcelona, Spain

L. Schio, L. Floreano,

CNR - Istituto Officina dei Materiali (IOM), TASC Laboratory , 34149 Trieste, Italy

V. Feyer and C. M. Schneider

Faculty of Physics and Center for Nanointegration Duisburg-Essen (CENIDE), University of Duisburg-Essen, 47048 Duisburg, Germany

C. M. Schneider

Department of Physics and Astronomy, UC Davis, Davis CA 95616, USA


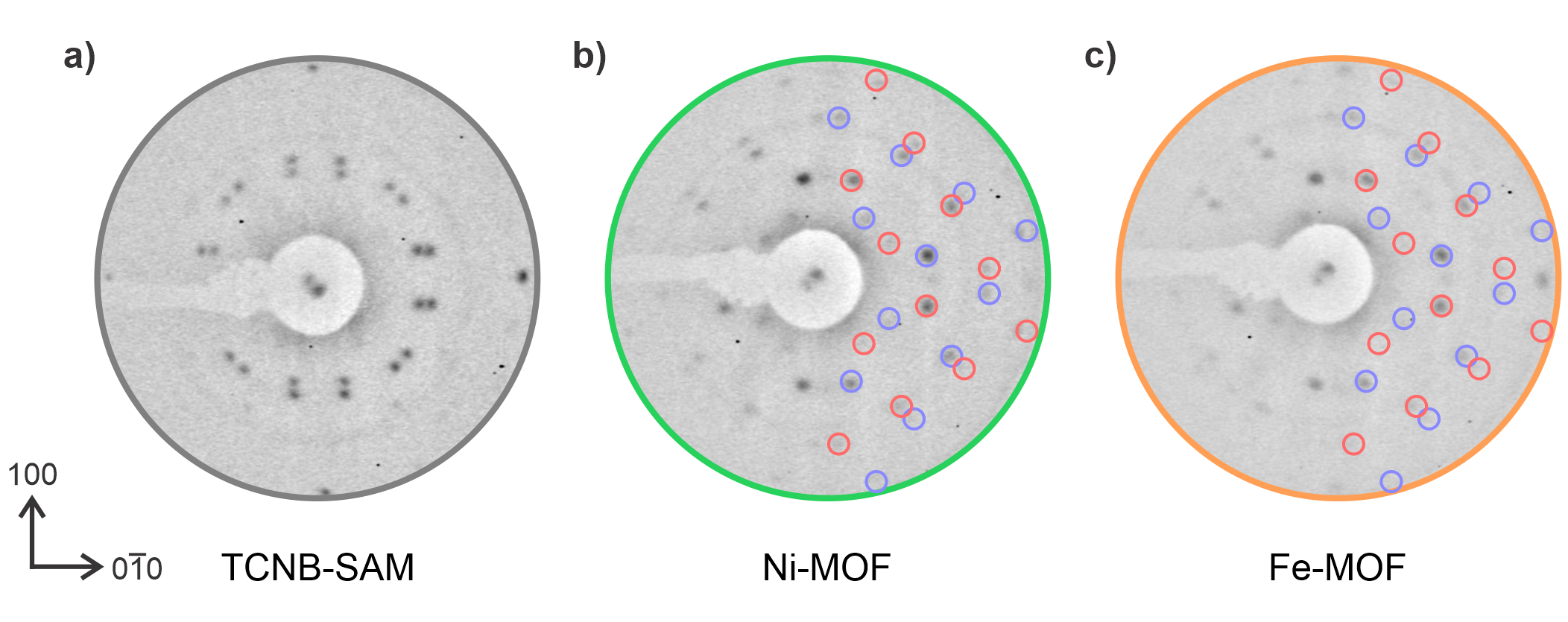


**Figure S1.** Experimental LEED patterns acquired with incident electron beam energy of 20 eV for the TCNB-SAM (a) and the Ni- (b) and Fe-MOF (c). The red and blue circles, corresponding to two rotational domains, indicate the LEED simulation patterns.


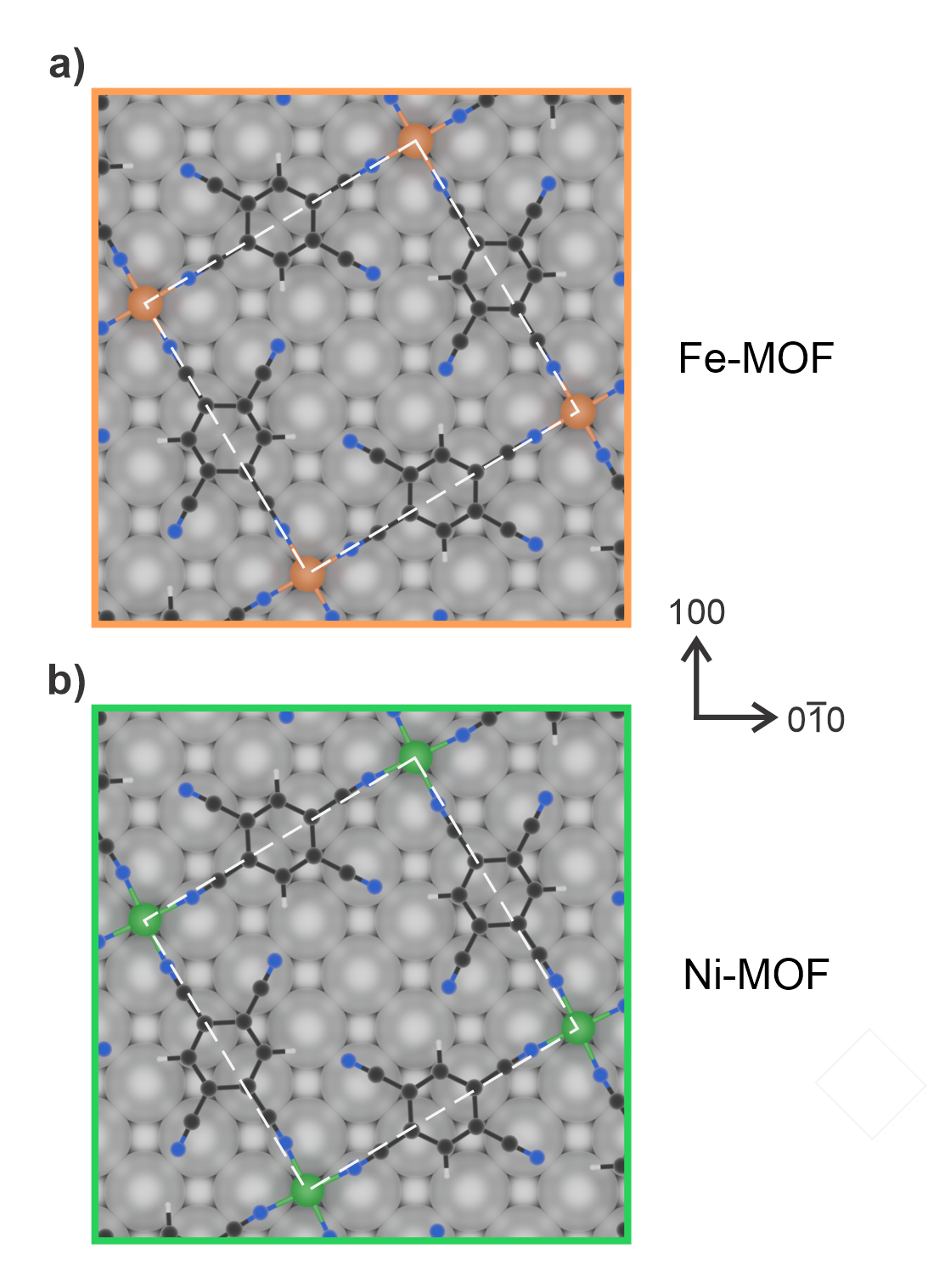


**Figure S2**. Real space structure representations of the relaxed structured for the Fe-MOF (a) and Ni-MOF (b) on Ag(100).


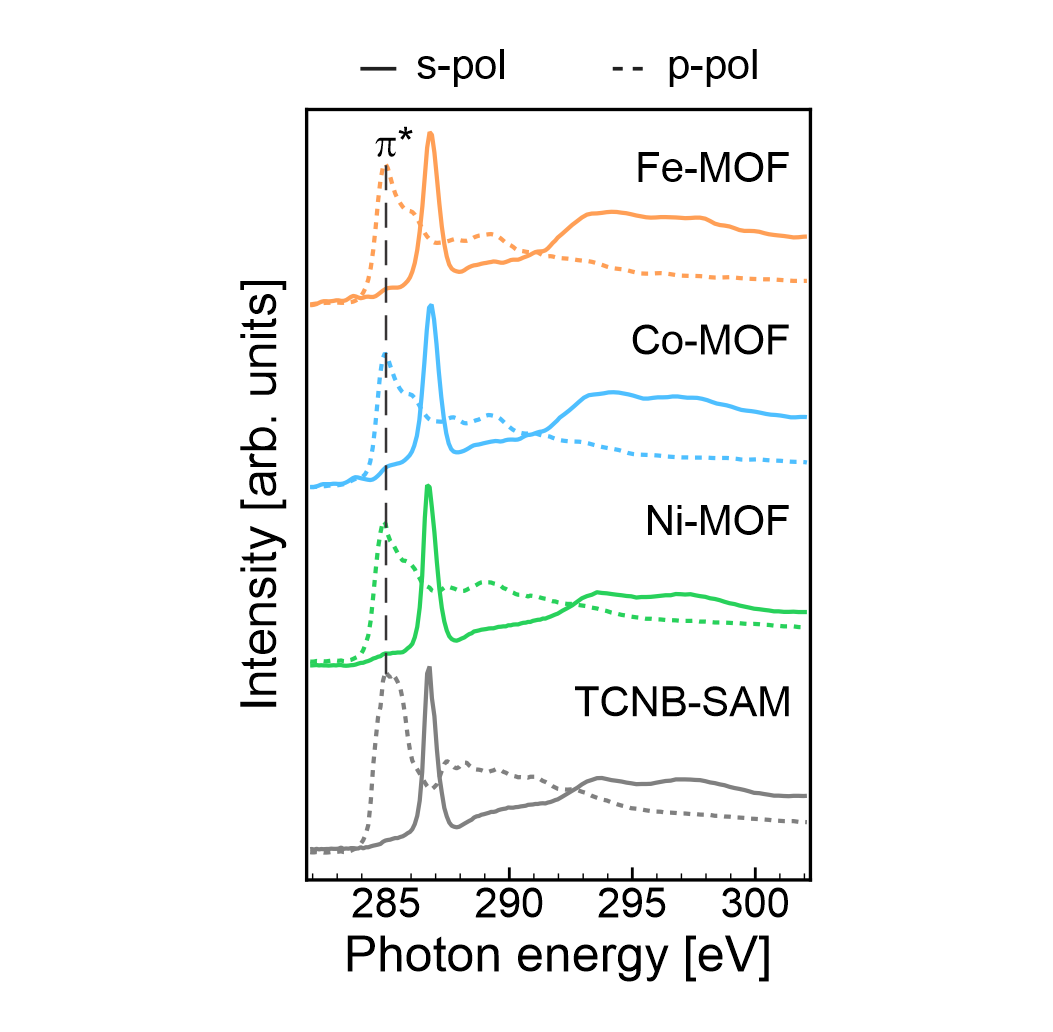


**Figure S3**. NEXAFS spectra acquired across the C K-edge with s- (solid line) and p-polarized light (dashed line). The following color code has been chosen: green, cyan and orange for the Ni-, Co- and Fe-MOFs and gray for the TCNB-SAM, respectively.


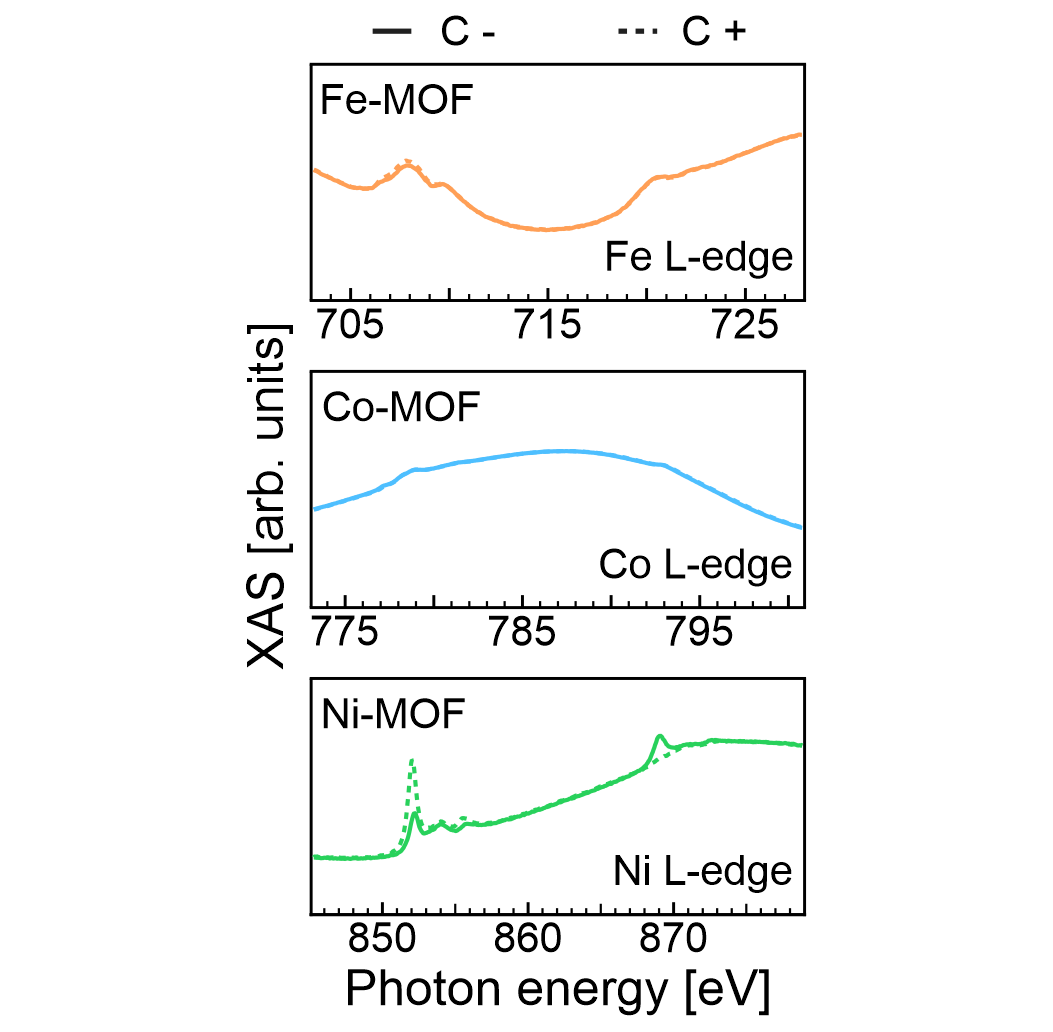


**Figure S4.** The x-ray absorption spectra acquired at a magnetic field B = 6 T along the wave vector of the incident photon with left and right circularly polarized light (solid and dashed lines, respectively) across the L_3_- and L_2_-edges of the TMs in the three studied systems: Ni-, Co- and Fe-MOF (green, cyan and orange).


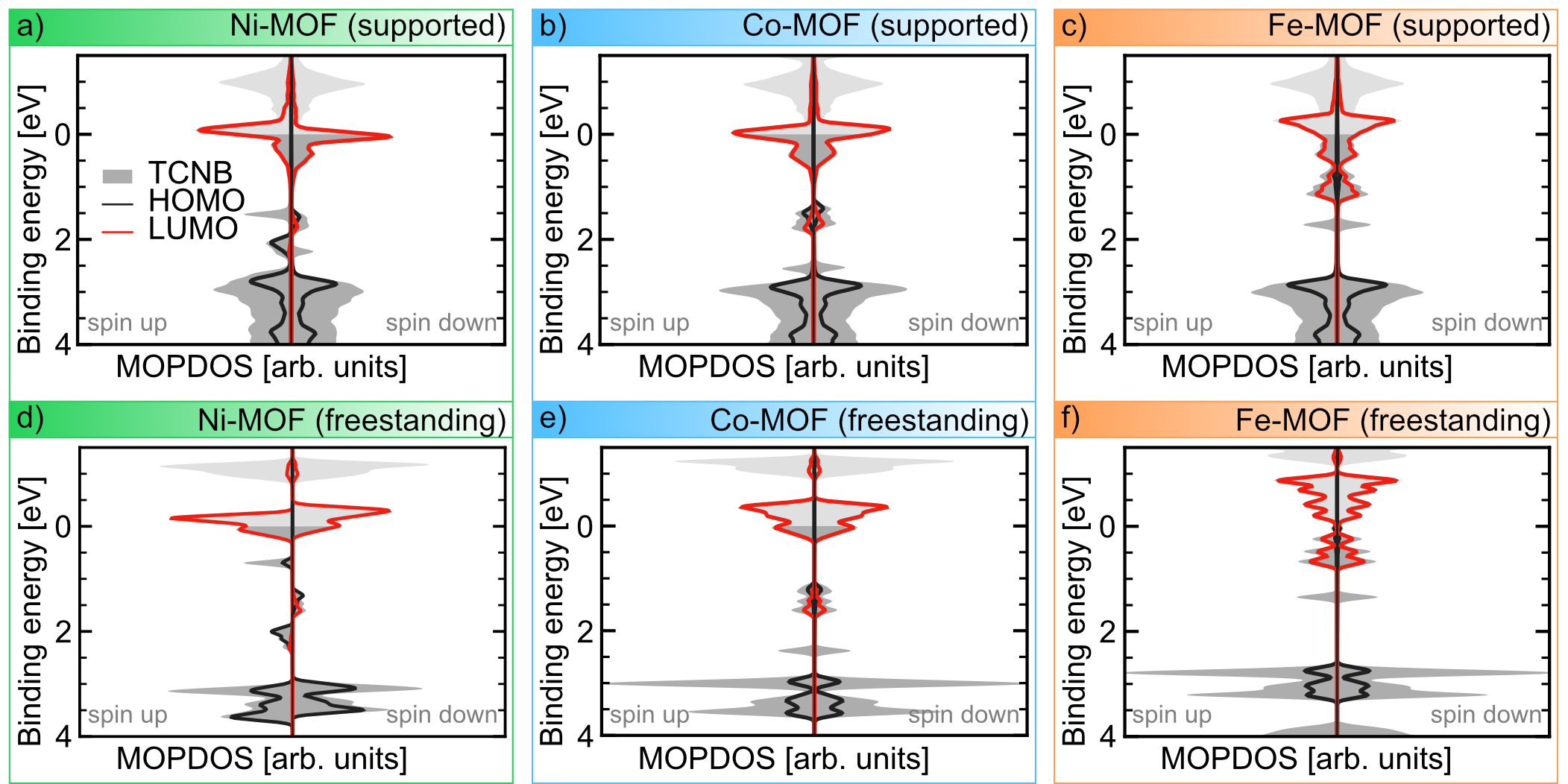


**Figure S5.** Density of states projected onto the molecular states of the TCNB molecules without transition metal or substrate (MOPDOS). The total projection onto all states is displayed as gray-shaded area with a darker gray below the Fermi edge. The projection onto the HOMO and LUMO orbitals only are shown as solid black and red line, respectively. a) Ni-MOF on Ag(100), b) Co-MOF on Ag(100), c) Fe-MOF on Ag(100), d) Ni-MOF as freestanding layer, e) Co-MOF as freestanding layer, f) Fe-MOF as freestanding layer.


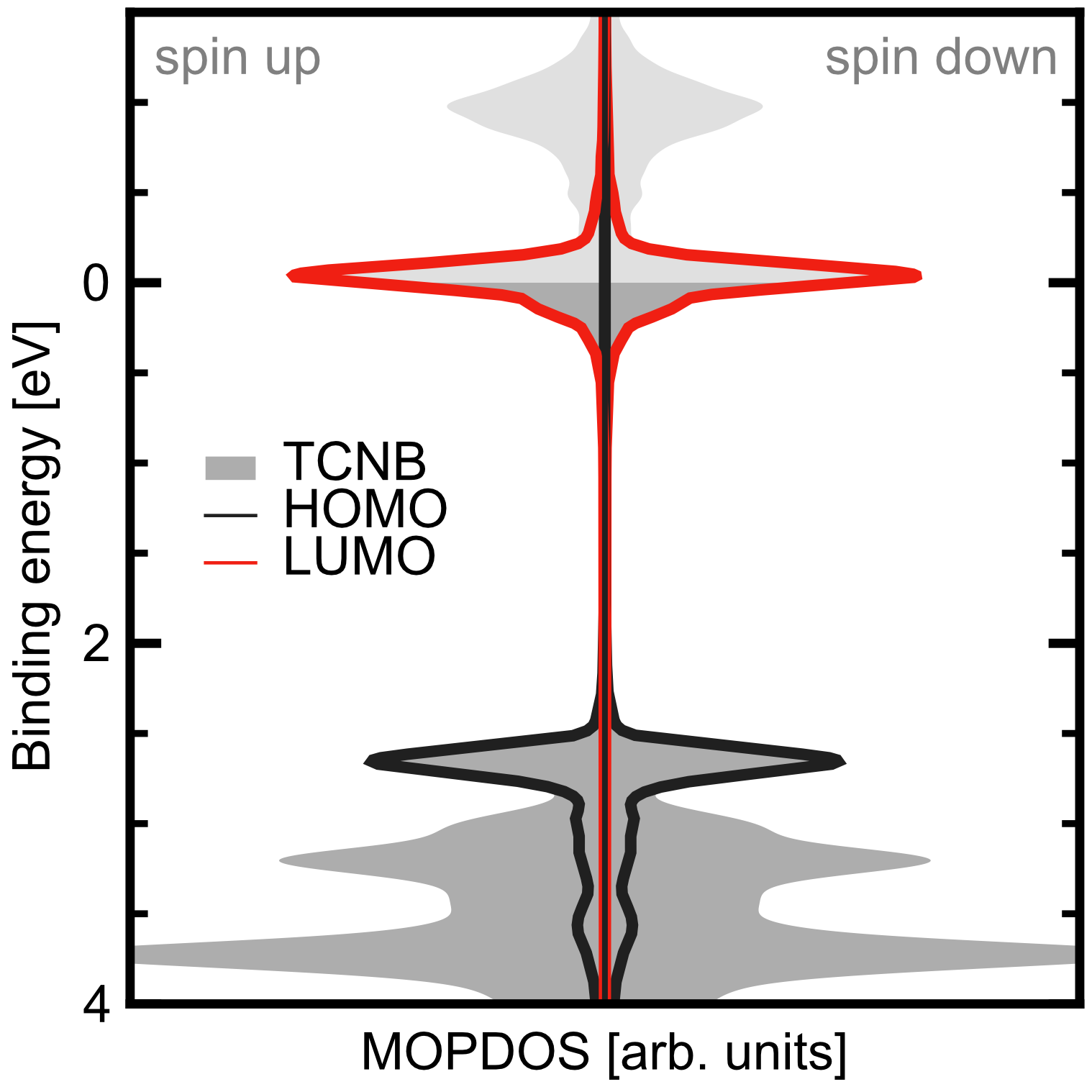


**Figure S6**. Density of states of a pristine layer of TCNB on Ag(100) projected onto the molecular states of TCNB (MOPDOS). The total projection onto all states is displayed as gray-shaded area with a darker gray below the Fermi edge. The projection onto the HOMO and LUMO orbitals only are shown as solid black and red line, respectively.


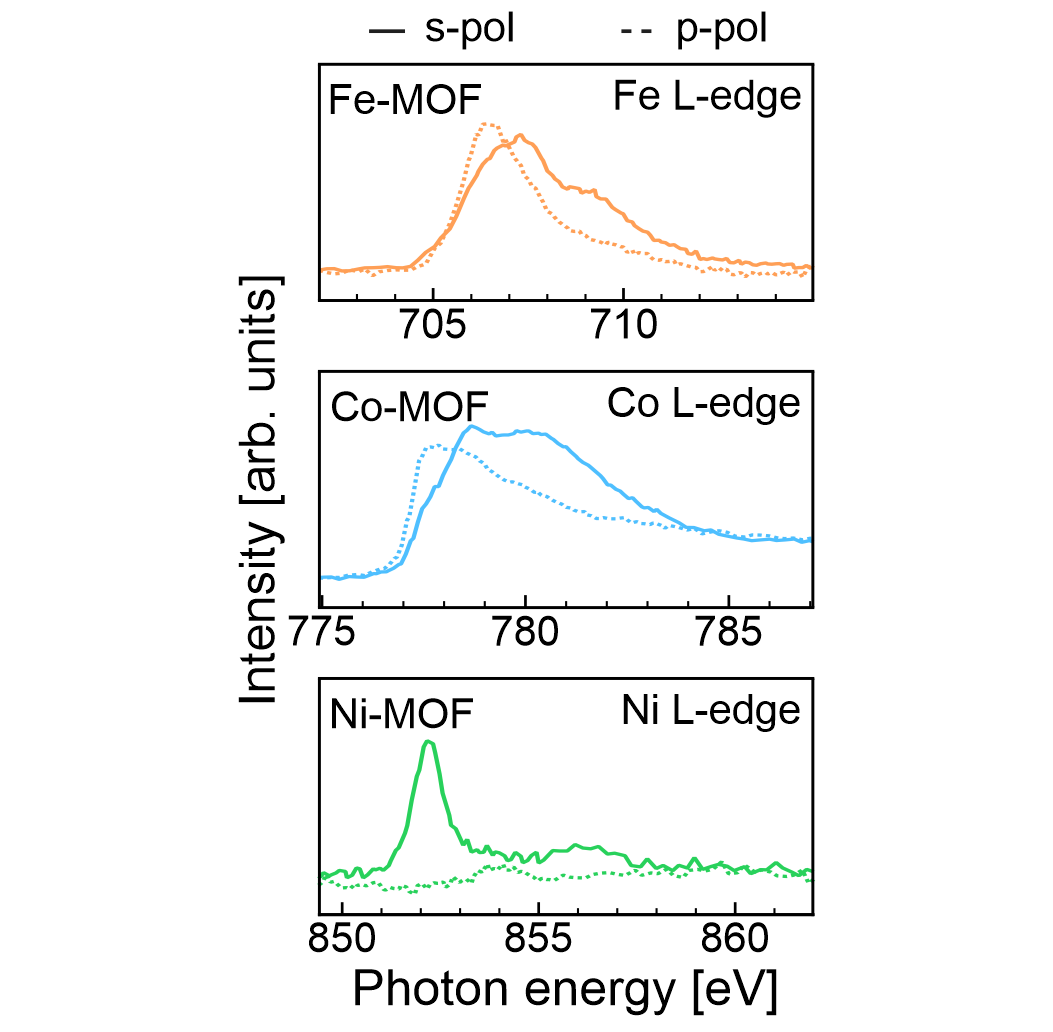


**Figure S7**. NEXAFS spectra acquired across the TM L_3_-edge with s- (solid line) and p-polarized light (dashed line) for the Fe-MOF (top), the Co-MOF (middle) and the Ni-MOF (bottom).


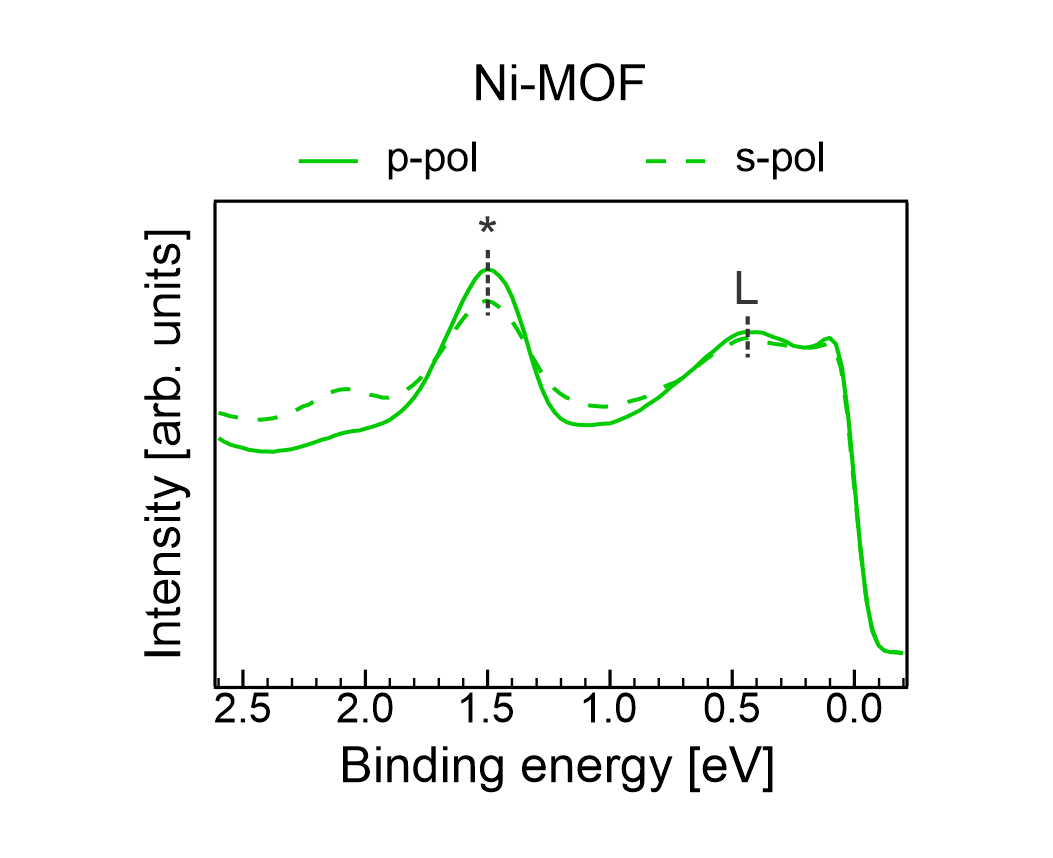


**Figure S8.** Valence band spectra obtained with an excitation photon energy of 30 eV and p- and s-polarized light (solid and dashed lines, respectively) for the Ni-MOF. "L-" and “*-” refer to the filled LUMO and the hybrid state.


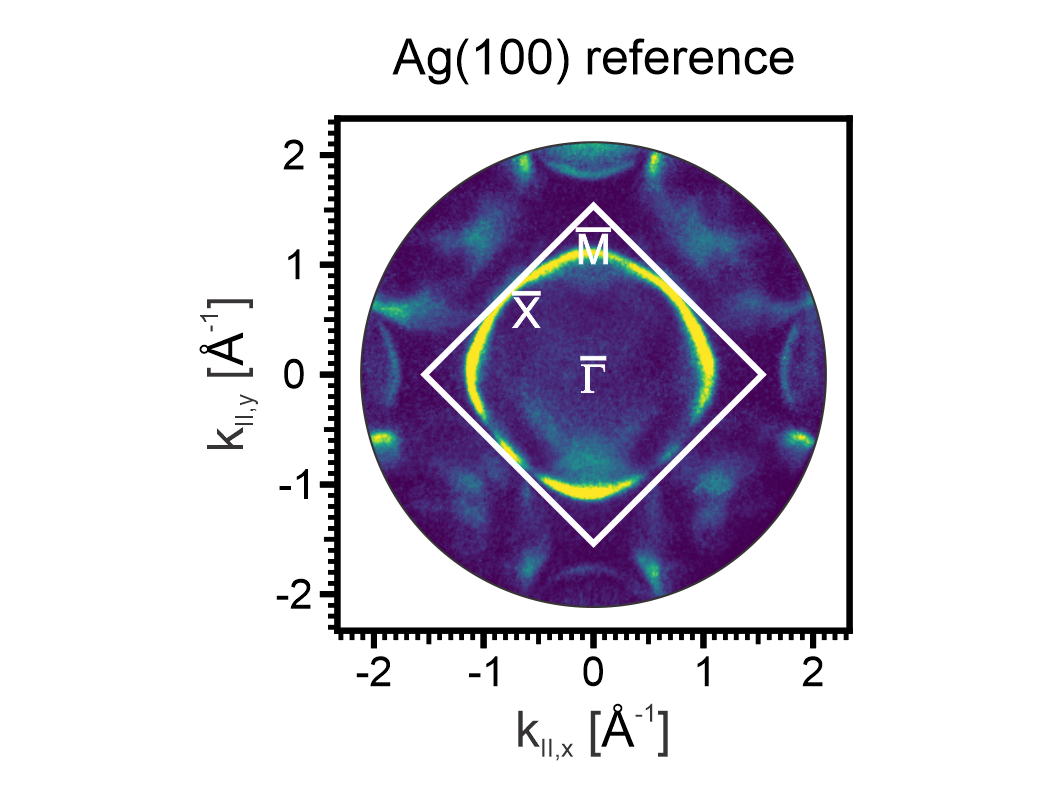


**Figure S9.** Constant energy 2D momentum map (k_||,x_, k_||,y_) of the clean Ag(100) substrate measured at about 1.4 eV of BE; the surface Brillouin zone is also reported.


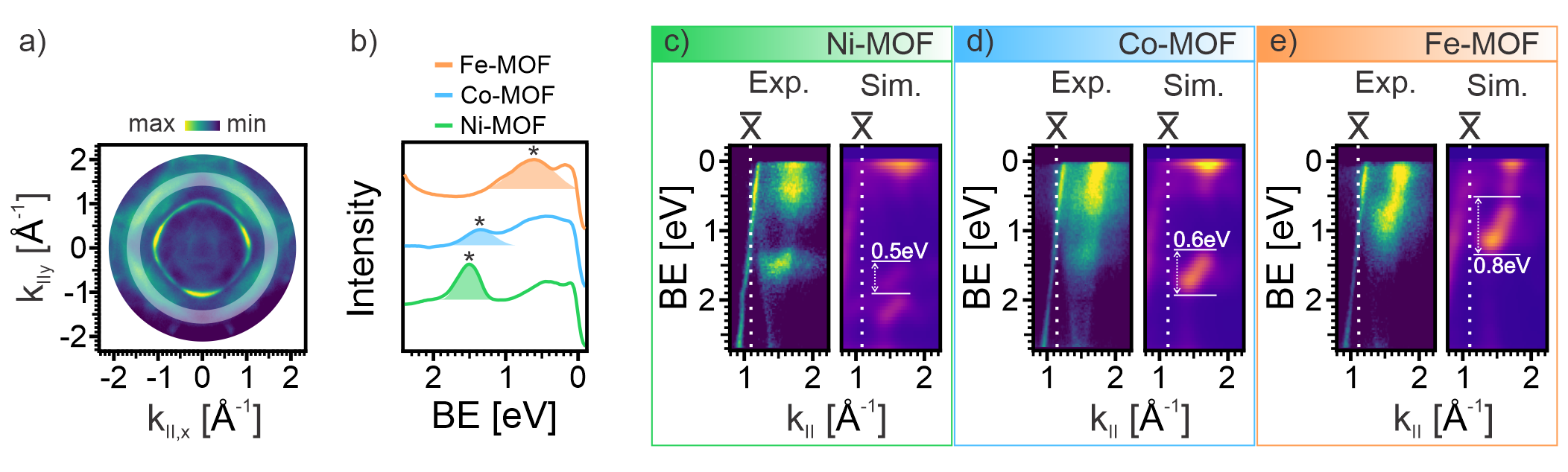


**Figure S10.** Evaluation of energy dispersion of the hybrid states (“*”) using the region of interest (ROI), represented by the shaded ring area in the momentum map in a). b) VB spectra extracted from the ROI. Green, cyan and orange lines refer to the Ni-, Co- and Fe-MOFs, respectively. c-e) Experimental (left) and simulated (right) valence band maps represented as BE vs momentum cuts along the $\bar{\Gamma}-\bar{X}$direction of the substrate: c) Ni-MOF, d) Co-MOF, e) Fe-MOF.
